# Supplementary material for: Skin basal cell carcinomas assemble a pro-tumorigenic spatially organized and self-propagating Trem2+ myeloid niche
Source: Nat Commun. 2023 May 10;14:2685. doi: 10.1038/s41467-023-37993-w (PMC10172319; doi:10.1038/s41467-023-37993-w)
Supplement: Supplementary file 1 — Supplementary Information [file 41467_2023_37993_MOESM1_ESM.pdf]

Supplementary Information:

Skin basal cell carcinomas assemble a pro-tumorigenic spatially organized and self-propagating Trem2+ myeloid niche

Daniel Haensel[1], Bence Daniel[2,3], Sadhana Gaddam[1], Cory Pan[1], Tania Fabo[1], Jeremy Bjelajac[1], Anna R. Jussila[1], Fernanda Gonzalez[1], Nancy Yanzhe Li[1], Yun Chen[4,5], JinChao Hou[4], Tiffany Patel[1], Sumaira Aasi[6], Ansuman T. Satpathy[2,3,7], and Anthony E. Oro[1,8]

[1]Program in Epithelial Biology, Stanford University School of Medicine, Stanford CA, United States of America
[2]Department of Pathology, Stanford University School of Medicine, Stanford CA, United States of America
[3]Gladstone-UCSF Institute of Genomic Immunology, San Francisco, CA 94158, USA
[4]Department of Pathology and Immunology, Washington University School of Medicine, St Louis, MO, United States of America
[5]Department of Neurology, Washington University School of Medicine, St Louis, MO, United States of America
[6]Department of Dermatology, Stanford University School of Medicine, Stanford CA, United States of America
[7]Parker Institute of Cancer Immunotherapy, San Francisco, CA 94305, USA.
[8]Correspondence to: Anthony E. Oro MD/PhD, Program in Epithelial Biology, Stanford University School of Medicine, Stanford CA, United States of America, Phone: +1 650-723-7843, Fax: +1 650-723-8762, E-mail: oro@stanford.edu

SUPPLEMENTARY FIGURE 1

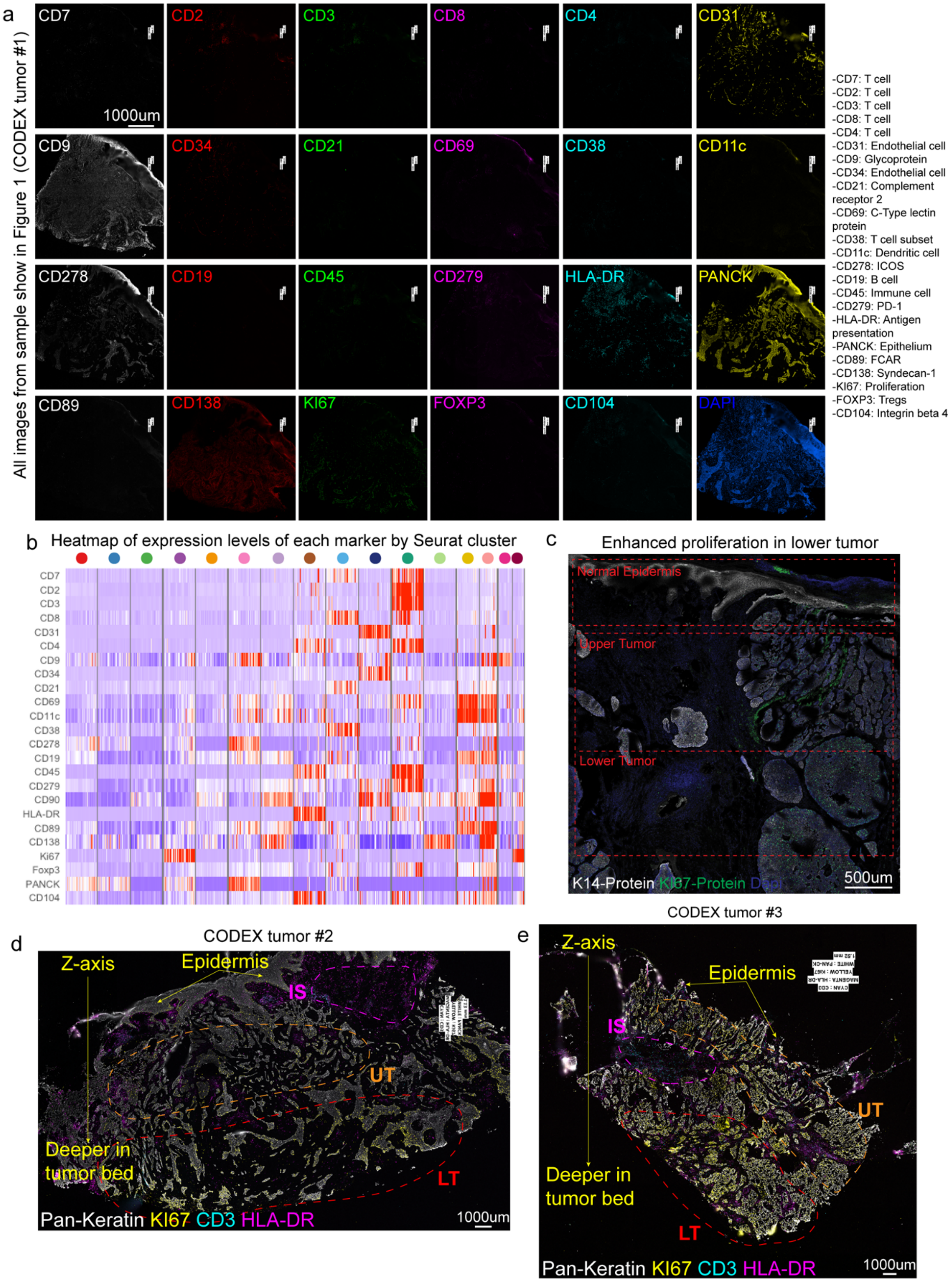

Supplementary Figure 1:

a. Single images of all the channels from each antibody for the image are represented in Figure 1.
b. Heatmap of the different levels of the markers in each of the clusters from Fig. 1b.
c. Additional immunoflourence staining examples (non-CODEX) for proliferation within human BCC tumors with Ki67 (green), K14 (white), and Dapi (blue).
d. CODEX tumor #2 example. The tumor epithelium and epidermis were labeled with Pan-Keratin (white), KI67 (yellow), CD3 (cyan), and HLA-DR (magenta). Cell fluoresce metadata for each marker was extracted and integrated with Seurat.
e. CODEX tumor #2 example. The tumor epithelium and epidermis were labeled with Pan-Keratin (white), KI67 (yellow), CD3 (cyan), and HLA-DR (magenta). Cell fluoresce metadata for each marker was extracted and integrated with Seurat.

Length of each scale bar is noted in figure. For a and c-e, representative data from a single biological replicate (n = 3 biological replicates collected from independent samples/patients).

SUPPLEMENTARY FIGURE 2

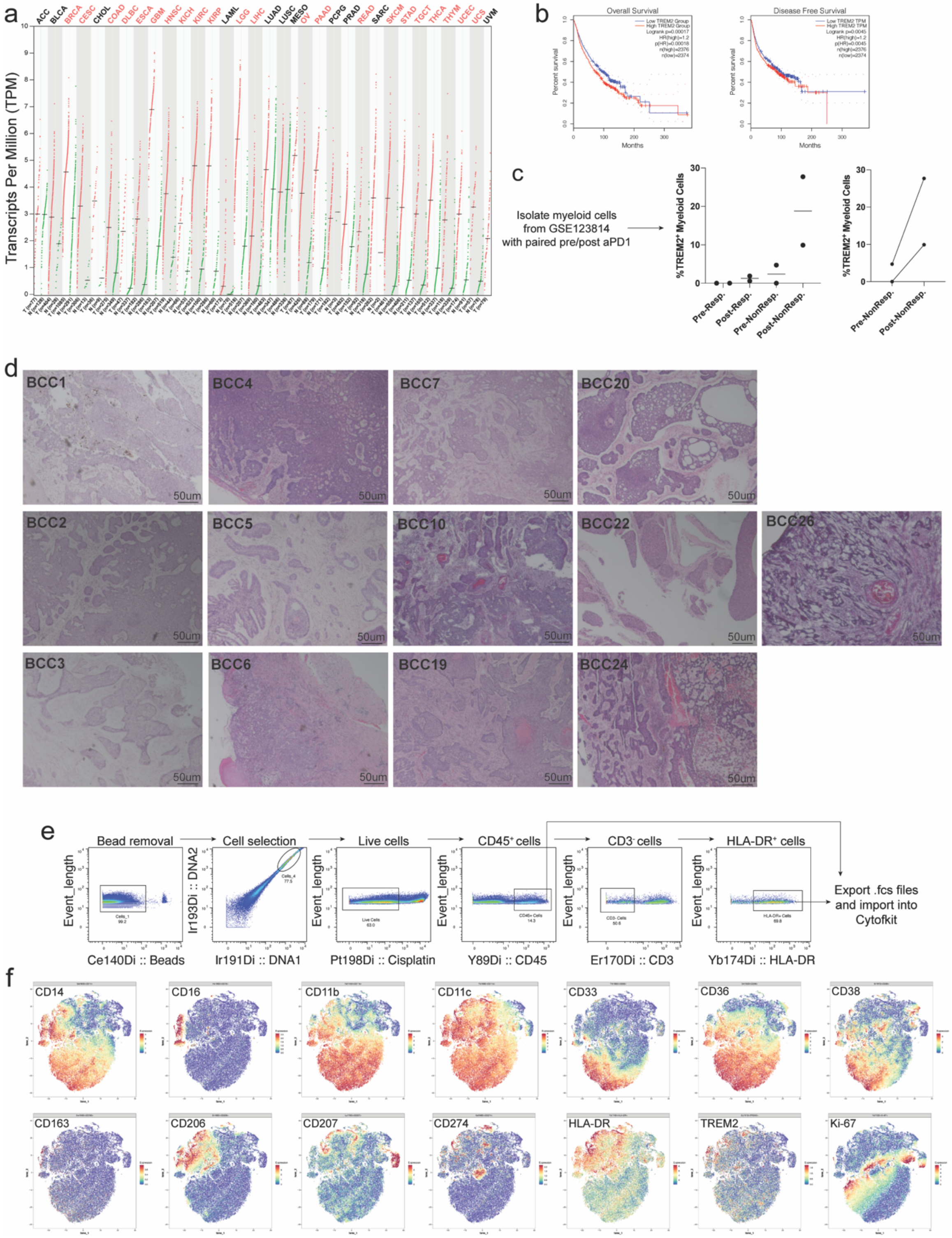

Supplementary Figure 2:

a. TREM2 expression levels in different types of tumors (red) relative to normal tissue (green).
b. Overall survival and disease-free survival for tumors with high and low expression of Trem2. Samples include all samples and tumor types (n = 2376 samples in 'High' TREM2 Group and n = 2374 samples in 'Low' TREM2 group) from Extended Fig. 2a. Survival curves are generated by GEPIA, which uses a log-rank test (or Mantel-Cox test).
c. Analysis of TREM2 cell percentage in responsive and non-response tumors pre- and post-aPD1 therapy (n = 2 independent tumors per group).
d. Histology of all 13 human BCC samples used for CyTOF analysis. Scale bar = 50 μm
e. CyTOF gating strategy to identify live $CD45^{+}$ immune cells and subsequent isolation of $CD3^{-}HLA\text{-}DR^{+}$ cells for downstream analysis
f. Feature plots of various indicated markers to characterize the $HLA\text{-}DR^{+}$ cells.

Source data are provided as a Source Data file.

SUPPLEMENTARY FIGURE 3

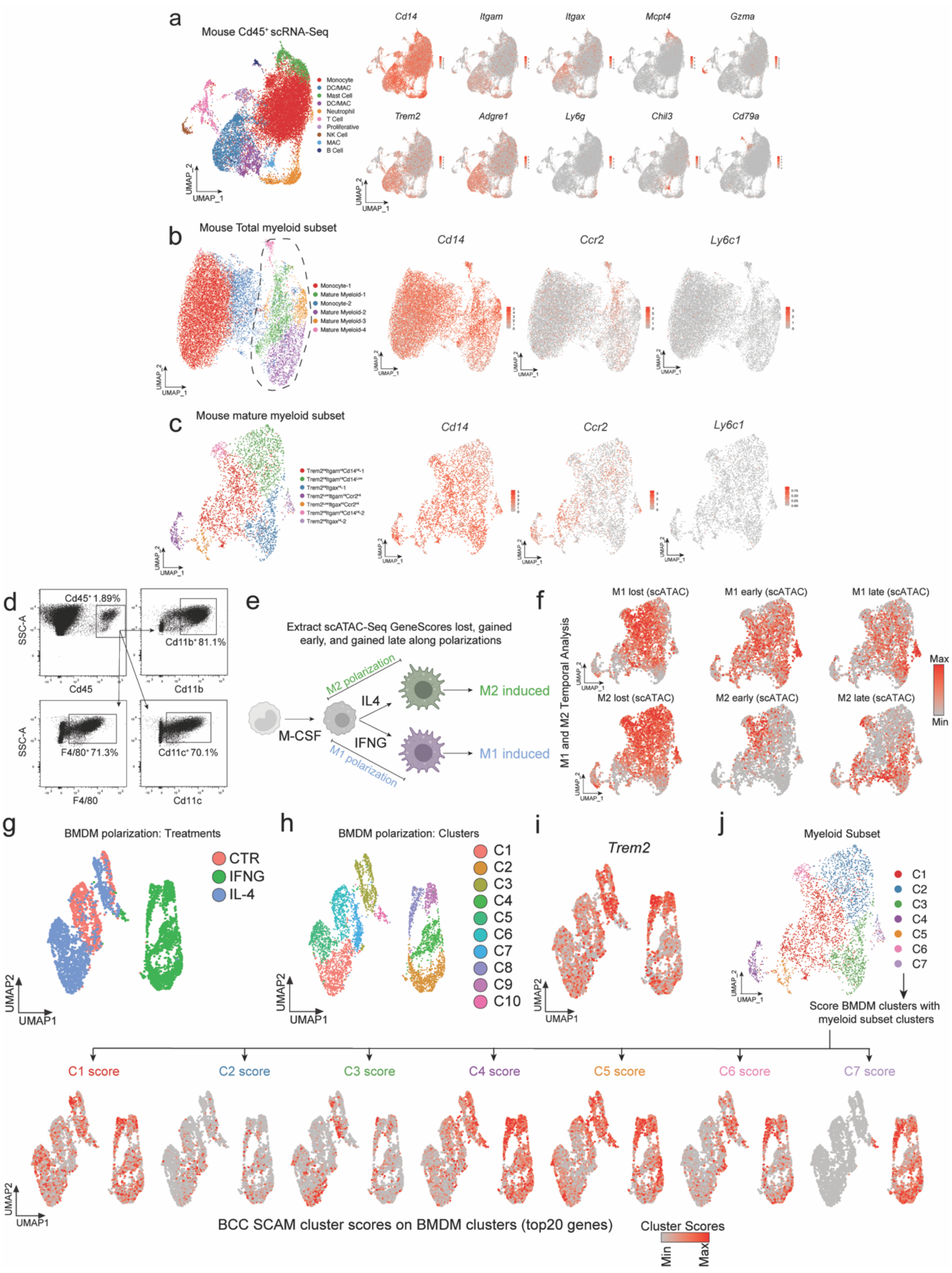

Supplementary Figure 3:

a. UMAP and subsequent feature plots of key markers of all Cd45$^+$ cells from mouse BCCs.
b. UMAP and subsequent feature plots of key markers of myeloid cells from mouse BCCs.
c. UMAP and subsequent feature plots of key markers of mature myeloid cells from mouse BCCs.
d. Flow cytometry gates for Trem2$^+$ myeloid cells, which quantification was shown in Fig. 4f.
e. Diagram of M1 and M2 polarization experiments to generate M1 and M2 polarization trajectories to determine genes that are lost, gained early, or gained late along the trajectory.
f. Feature plot of the M1- and M2-associated polarization trajectory scoring for the mBCC myeloid cells from Fig. 4c.
g. UMAP of scRNA-Seq analysis from control, IFNG, and IL-4 polarized BMDMs by experimental condition.
h. UMAP of scRNA-Seq analysis from control, IFNG, and IL-4 polarized BMDMs by cluster.
i. Feature plot of the mRNA level of *Trem2* for the UMAP shown in Extended Data Fig. 3g.
j. Scoring of BMDMs in Extended Data Fig. 3g by distinct mBCC cluster marker genes from Fig. 4c.

SUPPLEMENTARY FIGURE 4

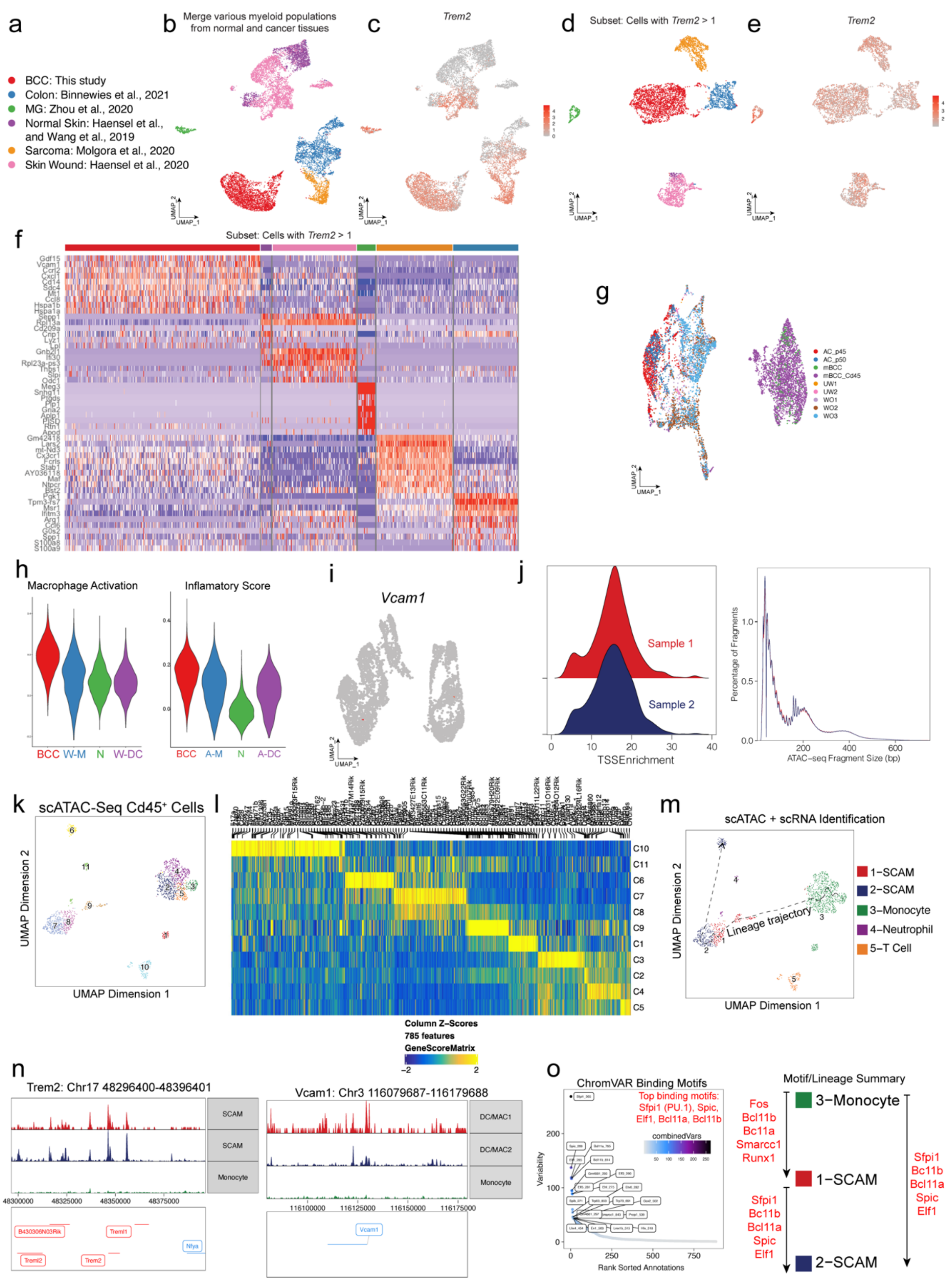

Supplementary Figure 4:

a. Overview and color labels of the different merged datasets are seen in Extended Data Fig. 4b.
b. UMAP of the merged myeloid fractions from each of the datasets outlined in Extended Data Fig. 4a.
c. Feature plot of *Trem2* for the plot shown in Extended Data Fig. 4b.
d. Subset UMAP of *Trem2*-expressing cells from Extended Data Fig. 4b.
e. Feature plot of *Trem2* for the plot shown in Extended Data Fig. 4d.
f. Heatmap showing the top marker genes of the various *Trem2*-expressing cells from the different datasets from Extended Data Fig. 4d.
g. UMAP of merged scRNA-Seq data as shown in Fig. 5a by sample ID.
h. Gene scoring of clusters in Fig. 5a for terms associated with macrophage activation and inflamation.
i. Feature plot of the level of *Vcam1* for the UMAP shown in Extended Data Fig. 3g.
j. TSS enrichment for the scATAC-Seq analysis of $Cd45^+$ cells from mouse BCCs.
k. UMAP plot of the different clusters for the scATAC-Seq analysis of $Cd45^+$ cells from mouse BCCs.
l. Heatmap of the associated marker genes that define each cluster.
m. UMAP plot of the integration of scRNA-Seq and scATAC-Seq analysis from mBCC SCAMs and myeloid cells. The predicted lineage trajectory is overlayed indicating a monocyte to SCAM maturation.
n. scATAC-Seq analysis of the peaks associated around *Trem2* and *Vcam1*.
o. Motifs from the scATAC-Seq are associated with the lineage transition from monocytes to SCAMs

SUPPLEMENTARY FIGURE 5

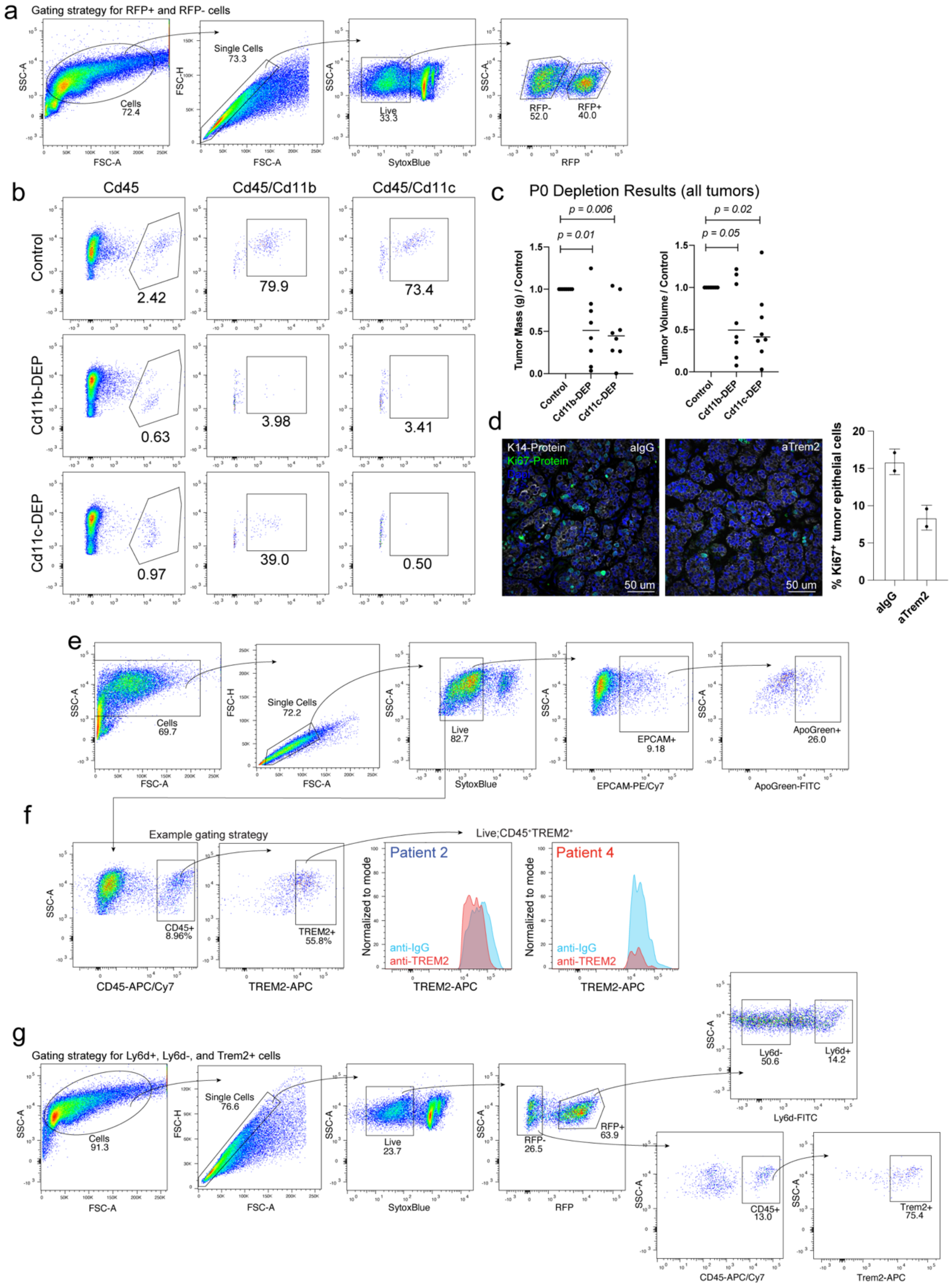

Supplementary Figure 5:

a. General flow cytometry gating strategy for RFP^+ and RFP- cells from the mouse BCC tumor model.
b. Additional flow cytometry analysis of column-mediated depletions in Fig. 6f.
c. Individual tumor sizes from column depletion transplantations in Fig. 6g (n = 8 individual tumors from mice). *p*-values were calculated using unpaired, two-tailed *t*-test.
d. Immunofluorescence staining for Ki67 (green), K14 (white), and Dapi (blue) in allografted tumors from mice treated with anti-IgG or anti-Trem2 (n = 2 individual tumors). Error bars represent mean +/- SD.
e. General flow cytometry gating strategy for PDOs.
f. General flow cytometry gating strategy to assess TREM2 levels on $TREM2^+$ cells from PDOs. Additional flow cytometry analysis for Patient 2 and Patient 4.
g. General flow cytometry gating strategy for SCAMs, $Ly6d^-$, and $Ly6d^+$ cells for organoid experiments in Fig. 6p-r.

Length of each scale bar is noted in figure. Source data are provided as a Source Data file.

SUPPLEMENTARY FIGURE 6

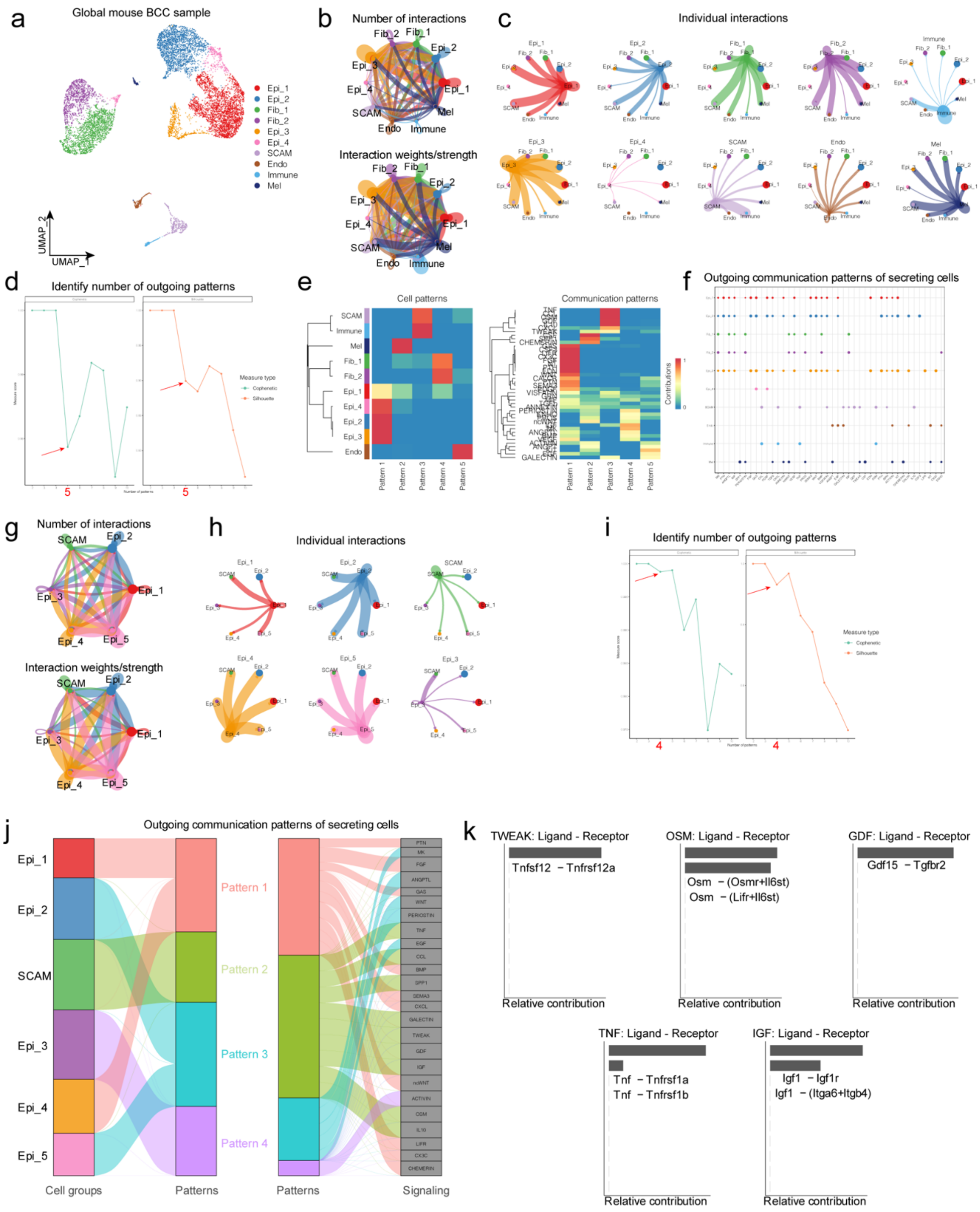

Supplementary Figure 6:

a. UMAP of the total tumor epithelium and stroma for CellChat analysis.
b. Interaction analysis between all components from Extended Data Fig. 7a.
c. Interaction analysis between individual components from Extended Data Fig. 7a.
d. Identification of the number of outgoing patterns for CellChat analysis for the population in Extended Data Fig. 7a.
e. Clustering of the distinct Cell and Communication patterns between the total tumor epithelium and stroma from Extended Data Fig. 7a.
f. CellChat outgoing communication patterns overview.
g. Interaction analysis between all components from Fig. 7a.
h. Interaction analysis between individual components from Fig. 7a.
i. Identification of the number of outgoing patterns for CellChat analysis for the population in Fig. 7a.
j. Additional pattern analysis for outgoing communications for populations in Fig. 7a.
k. Identification of the specific ligands and receptors predicated to mediate communication between SCAMs and the tumor epithelium from Fig. 7a.

SUPPLEMENTARY FIGURE 7

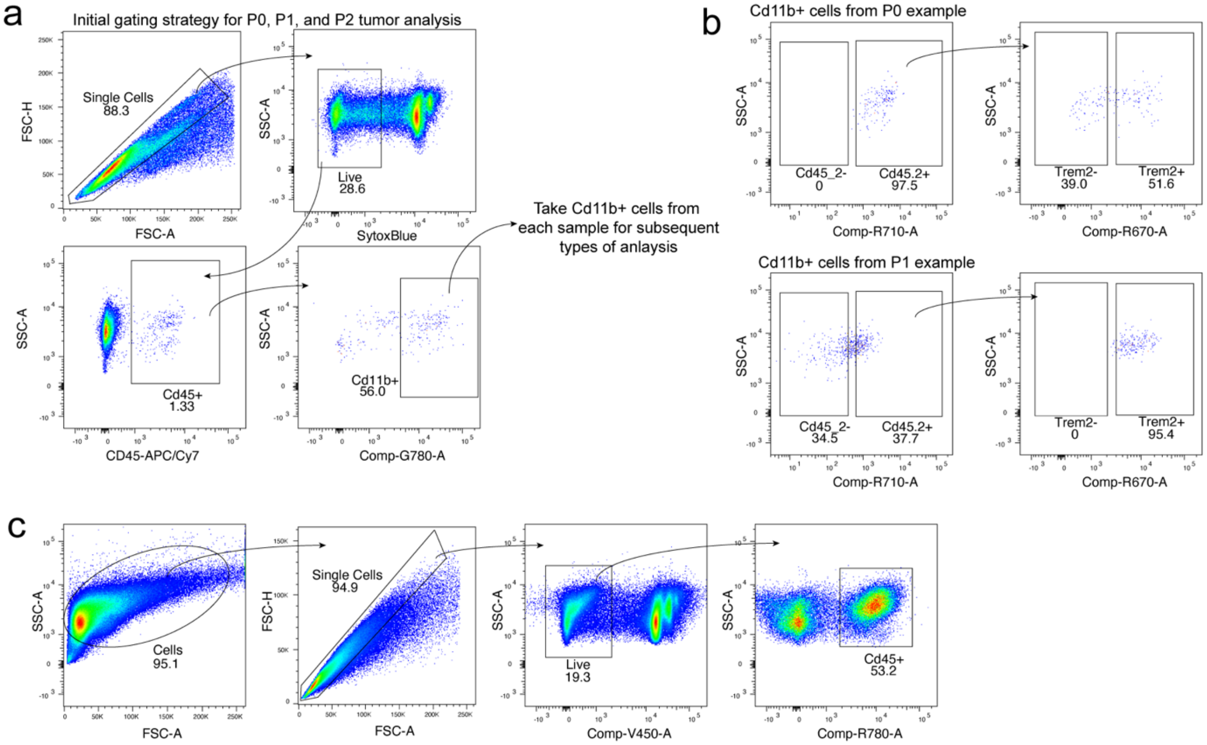

Supplementary Figure 7:

a. Additional general flow cytometry gating strategy for $Cd11b^+$ cells within the P0, P1, and P2 tumors in Fig. 8d.
b. Additional general flow cytometry gating strategy to distinguish CD45.2+ and Cd45.2- cells and their subsequent levels of Trem2 in Fig. 8e.
c. Additional general flow cytometry gating strategy for GFP+ monocytes in Fig. 8l.
