# Supplementary material for: Skin basal cell carcinomas assemble a pro-tumorigenic spatially organized and self-propagating Trem2+ myeloid niche
Source: Nat Commun. 2023 May 10;14:2685. doi: 10.1038/s41467-023-37993-w (PMC10172319; doi:10.1038/s41467-023-37993-w)
Supplement: Supplementary file 3 — Description of Additional Supplementary Files [file 41467_2023_37993_MOESM3_ESM.pdf]

Description of additional supplementary files

Supplementary Data 1: Marker genes from the scRNA-Seq of the *HLA-DRA*$^{+}$ clusters from the human BCC (Fig. 2c). Markers identified through Seurat's 'FindAllMarkers' function, which identifies differentially expressed genes between clusters via a Wilcoxon Rank Sum test.

Supplementary Data 2: Marker genes from the scRNA-Seq of the sorted Cd45^{+} clusters from the mouse BCC (Fig. 4a). Markers identified through Seurat's 'FindAllMarkers' function, which identifies differentially expressed genes between clusters via a Wilcoxon Rank Sum test.

Supplementary Data 3: Marker genes from the mature myeloid fraction clusters from the scRNA-Seq of the sorted Cd45^{+} cells (Fig. 4c). Markers identified through Seurat's 'FindAllMarkers' function, which identifies differentially expressed genes between clusters via a Wilcoxon Rank Sum test.

Supplementary Data 4: Marker genes of the total merged populations of myeloid cells (Supplementary Fig. 4a). Markers identified through Seurat's 'FindAllMarkers' function, which identifies differentially expressed genes between clusters via a Wilcoxon Rank Sum test.

Supplementary Data 5: Marker genes of the merged myeloid populations from normal skin, wounded skin, and BCC (Fig. 5a). Markers identified through Seurat's 'FindAllMarkers' function, which identifies differentially expressed genes between clusters via a Wilcoxon Rank Sum test.

Supplementary Data 6: DESeq results from ATAC-Seq analysis comparing Trem2^{+} cells from wounds and normal skin. DESeq uses a negative binomial distribution model.

Supplementary Data 7: DESeq results from ATAC-Seq analysis comparing Trem2^{+} cells from BCCs and normal skin. DESeq uses a negative binomial distribution model.

Supplementary Data 8: DESeq results from ATAC-Seq analysis comparing Trem2^{+} cells from BCCs and wounds. DESeq uses a negative binomial distribution model.

Supplementary Data 9: Differential AP-1 motif analysis between Trem2^{+} cells from BCCs and normal skin. DESeq uses a negative binomial distribution model.

Supplementary Data 10: Marker genes from the scRNA-Seq of the merged CD45.2^{+} and CD45.2^{-} fractions. Markers identified through Seurat's 'FindAllMarkers' function, which identifies differentially expressed genes between clusters via a Wilcoxon Rank Sum test.
