# Supplementary material for: Skin basal cell carcinomas assemble a pro-tumorigenic spatially organized and self-propagating Trem2+ myeloid niche
Source: Nat Commun. 2023 May 10;14:2685. doi: 10.1038/s41467-023-37993-w (PMC10172319; doi:10.1038/s41467-023-37993-w)
Supplement: Supplementary file 14 — Reporting Summary [file 41467_2023_37993_MOESM14_ESM.pdf]

nature portfolio

Corresponding author(s): Anthony Oro

Last updated by author(s): Mar 29, 2023

Reporting Summary

Nature Portfolio wishes to improve the reproducibility of the work that we publish. This form provides structure for consistency and transparency in reporting. For further information on Nature Portfolio policies, see our Editorial Policies and the Editorial Policy Checklist.

Statistics

For all statistical analyses, confirm that the following items are present in the figure legend, table legend, main text, or Methods section.

n/a	Confirmed	
☐	☒	The exact sample size (*n*) for each experimental group/condition, given as a discrete number and unit of measurement
☐	☒	A statement on whether measurements were taken from distinct samples or whether the same sample was measured repeatedly
☐	☒	The statistical test(s) used AND whether they are one- or two-sided<br>*Only common tests should be described solely by name; describe more complex techniques in the Methods section.*
☒	☐	A description of all covariates tested
☐	☒	A description of any assumptions or corrections, such as tests of normality and adjustment for multiple comparisons
☐	☒	A full description of the statistical parameters including central tendency (e.g. means) or other basic estimates (e.g. regression coefficient) AND variation (e.g. standard deviation) or associated estimates of uncertainty (e.g. confidence intervals)
☐	☒	For null hypothesis testing, the test statistic (e.g. *F*, *t*, *r*) with confidence intervals, effect sizes, degrees of freedom and *P* value noted<br>*Give P values as exact values whenever suitable.*
☒	☐	For Bayesian analysis, information on the choice of priors and Markov chain Monte Carlo settings
☒	☐	For hierarchical and complex designs, identification of the appropriate level for tests and full reporting of outcomes
☒	☐	Estimates of effect sizes (e.g. Cohen's *d*, Pearson's *r*), indicating how they were calculated

Our web collection on statistics for biologists contains articles on many of the points above.

Software and code

Policy information about availability of computer code

Data collection	The various commercial/open source software was used for data collection: BD FACSDiva v8.0.1, Lecia LAS X v3.7.6, Illumina HiSeq and NextSeq System Suite, CODEX Instrument Manager v1.29, and the Keyence BZ-X software.
Data analysis	The various commercial/open source software was used for data analysis: Seurat v3, FIJI 2, 10x Genomics Cell Ranger v3.1.0, R v4.2.1, Monocle 2 v2.14.0, ArchR v1.0.1, CODEX Multiplex Analysis Viewer, Bowtie2 v2.3.4.1, Samtools v1.8, DESeq2 v1.38.3, FlowJo v10.6, CellChat v1.1.3, and HOMER v4.11.

For manuscripts utilizing custom algorithms or software that are central to the research but not yet described in published literature, software must be made available to editors and reviewers. We strongly encourage code deposition in a community repository (e.g. GitHub). See the Nature Portfolio guidelines for submitting code & software for further information.

Data

Policy information about availability of data

All manuscripts must include a data availability statement. This statement should provide the following information, where applicable:

- Accession codes, unique identifiers, or web links for publicly available datasets
- A description of any restrictions on data availability
- For clinical datasets or third party data, please ensure that the statement adheres to our policy

The human specific sequencing generated from this study has been deposited in the dbGaP database under the accession code phs003242.v1.p1. All new sequencing data have been deposited in GSE204952. Previous human BCC scRNA-Seq data used in this study are available in dbGAP database under the accession

code phs003103.v1.p1. Previous mouse BCC scRNA-Seq data used in this study are available in the NCBI Gene Expression Omnibus under the SuperSeries code: GSE186184. The resistant human BCC scRNA-Seq publicly available data used in this study are available in the NCBI Gene Expression Omnibus under the accession code: GSE123814. Previous mouse colon cancer scRNA-Seq data used in this study are available in the NCBI Gene Expression Omnibus under the code: GSE165404. The mouse microglia scRNA-Seq data used in this study are available in the NCBI Gene Expression Omnibus under the SuperSeries code: GSE140511. The mouse normal skin scRNA-Seq data used in this study are available in the NCBI Gene Expression Omnibus under the accession codes: GSE142471 and GSE108709. The mouse wound scRNA-Seq data used in this study are available in the NCBI Gene Expression Omnibus under the accession code: GSE142471. The mouse sarcoma scRNA-Seq used in this study are available in the NCBI Gene Expression Omnibus under the accession code: GSE151710. The data relating to TREM2 expression in various cancer types can be accessed through the GEPIA web interface (http://gepia.cancer-pku.cn/).

Human research participants

Policy information about studies involving human research participants and Sex and Gender in Research.

Reporting on sex and gender	All patient samples have been completely de-identified as per IRB protocols and we do not have this information.
Population characteristics	All patient samples have been completely de-identified as per IRB protocols and we do not have this information.
Recruitment	Patients are asked by physician if they would like to participate at time. There is no obvious self-selection bias or other biases that are present.
Ethics oversight	All patient samples were obtained through written informed consent and subsequently de-identified. All protocols for sample acquisition and usage are in accordance with the reviewed protocol by the Stanford University Institutional Review Board, protocol #18325 (Stanford, CA).

Note that full information on the approval of the study protocol must also be provided in the manuscript.

Field-specific reporting

Please select the one below that is the best fit for your research. If you are not sure, read the appropriate sections before making your selection.

☒ Life sciences ☐ Behavioural & social sciences ☐ Ecological, evolutionary & environmental sciences

For a reference copy of the document with all sections, see nature.com/documents/nr-reporting-summary-flat.pdf

Life sciences study design

All studies must disclose on these points even when the disclosure is negative.

Sample size	For all experiments requiring statistical analysis, we aimed to have a minimum of n = 3 biological replicates in order to derive p-values. We noted exact sizes either within the methods section or the figure legends. Sample size selection was based on similar types of experiments in the literature.
Data exclusions	No data was excluded from analysis.
Replication	All experiments were repeated at a minimum of 2 times will all replication attempts being successful.
Randomization	For all relevant studies/experiments, the same homogeneous sample was randomly split into either a treatment or control groups.
Blinding	Investigators were blind to primary human samples that were acquired for sequencing experiments as they were de-identified. For the various experimental/treatment studies, investigator was not blinded to group allocation because the same investigator conducted data collection and data analysis.

Reporting for specific materials, systems and methods

We require information from authors about some types of materials, experimental systems and methods used in many studies. Here, indicate whether each material, system or method listed is relevant to your study. If you are not sure if a list item applies to your research, read the appropriate section before selecting a response.

Materials & experimental systems

n/a	Involved in the study	
	x	Antibodies
	x	Eukaryotic cell lines
x		Palaeontology and archaeology
	x	Animals and other organisms
x		Clinical data
x		Dual use research of concern

Methods

n/a	Involved in the study	
x		ChIP-seq
	x	Flow cytometry
x		MRI-based neuroimaging

Antibodies

Antibodies used

1. APC anti-human/mouse TREM2 (R&D; FAB17291A; Clone: 237920)
2. APC/Cy7 anti-mouse CD45 (BioLegend; 103116; Clone: 30-F11)
3. Alexa700 anti-mouse CD45.2 (BioLegend; 109822; Clone: 104)
4. PerCP/Cy5 anti-mouse CD11b (BioLegend; 101228; Clone M1/70)
5. BV605 anti-mouse Cd11c (BioLegend; 117334; Clone N418)
6. FITC anti-mouse Ly6d (BioLegend; 138606; Clone: 49-H4)
7. PE/Cy7 anti-mouse Vcam1 (BioLegend; 105720; Clone: 429)
8. BC510 anti-mouse F4/80 (BioLegend; 123135; Clone: BM8)
9. PE/Cy7 anti-human CD326 (BioLegend; 324222; Clone: 9C4)
10. PE anti-human VCAM1 (BioLegend; 305805; Clone: STA)
11. CD2-BX002-550 (Akoya; 4250005; Clone: RPA-2.10)
12. CD3-BX015-Cy5 (Akoya; 4350008; Clone: UCHT1)
13. CD4-BX021-Cy5 (Akoya; 4350010; Clone: SK3)
14. CD5-BX024-Cy5 (Akoya; 4350011; Clone: UCHT2)
15. CD7-BX025-488 (Akoya; 4450022; Clone: CD7-6B7)
16. CD8-BX004-488 (Akoya; 4150004; Clone: SK1)
17. CD9-BX028-488 (Akoya; 4450016; Clone: H19a)
18. Cd11c-BX027-Cy5 (Akoya; 4350012; Clone: S-HCL-3)
19. CD19-BX003-Cy5 (Akoya; 4350003; Clone: H1B19)
20. CD21-BX013-488 (Akoya; 4450009; Clone: Bu32)
21. CD31-BX032-550 (Akoya; 4250009; Clone: WM59)
22. CD34-BX035-550 (Akoya; 4250020; Clone: 561)
23. CD38-BX007-488 (Akoya; 4150007; Clone: HB-7)
24. CD45-BX001-488 (Akoya; 4150003; Clone: HI30)
25. CD69-BX041-550 (Akoya; 4250022; Clone: FN50)
26. CD89-BX030-Cy5 (Akoya; 4350017; Clone: A59)
27. CD90-BX022-488 (Akoya; 4150021; Clone: 5E10)
28. CD104-BX005-550 (Akoya; 4250008; Clone: 58XB4)
29. CD138-BX010-488 (Akoya; 4150008: Clone MI15)
30. CD278-BX017-550 (Akoya; 4250013; Clone: C398.4A)
31. CD279-BX014-550 (Akoya; 4250010; Clone: EH12.2H7)
32. HLA-DR-BX026-550 (Akoya; 4250006; Clone: AKYP0017)
33. Ki67-BX047-550 (Akoya; 4250019; Clone: B56)
34. PANCK-BX019-488 (Akoya; 4150020; Clone: AE-1/AE-3)
35. CD19-142Nd (Fluidigm; 201317; Clone: HIB19)
36. Cd11b-144Nd (Fluidigm; 201317; Clone: ICRF44)
37. CD7-147Sm (Fluidigm; 201317; Clone: CD7-6B7)
38. CD66-149Sm (Fluidigm; 201317; Clone: CD66a-B1.1)
39. CD36-152Sm (Fluidigm; 201317; Clone: 5-271)
40. CD163-154Sm (Fluidigm; 201317; Clone: GH/61)
41. CD11c-159Tb (Fluidigm; 201317; Clone: Bu15)
42. CD14-160Gd (Fluidigm; 201317; Clone: M5E2)
43. CD16-165Ho (Fluidigm; 201317; Clone: 3G8)
44. CD38-167Er (Fluidigm; 201317; Clone: HIT2)
45. CD206-168Er (Fluidigm; 201317; Clone: 15-2)
46. CD33-169Tm (Fluidigm; 201317; Clone: WM53)
47. CD3-170Er (Fluidigm; 201317; Clone: UCHT1)
48. HLA-DR-174Yb (Fluidigm; 201317; Clone: L243)
49. CD45-89Y (Fluidigm; 3089003B; Clone: HI30)
50. CD207-175Lu (Fluidigm; 3175016B; Clone: 4C7)
51. CD279-155Gd (Fluidigm; 3155009B; Clone: EH12.2H7)
52. CD8a-146Nd (Fluidigm; 3146001B; Clone: RPA-T8)
53. TIM-3-153Eu (Fluidigm; 3153008B; Clone: F38-2E2)
54. TIGIT-209Bi (Fluidigm; 3209013B; Clone: MBSA43)

55. CD274-156Gd (Fluidigm; 3156026B; Clone: 29E.2A3)
56. TREM2 antibody (R&D Systems; MAB17291-500; Clone: 237920)
57. Human IgG4 antibody (R&D Systems; MAB9895-100; Clone: 985547)
58. Human PD1 antibody (Selleckchem; A2002; Clone: ONO-4538)
59. Mouse Osm antibody (R&D Systems; AF-495-NA)
60. Mouse IgG2b isotype control antibody (BioXCell; BP0086; Clone: MPC-11)
61. Chicken K14 antibody (BioLegend; SIG-3476-100; Clone: Poly9060)
62. Rabbit Ki67 antibody (Abcam; ab16667; Clone SP6)
63. Mouse HLA-DR antibody (Abcam; ab136320; Clone: L243)
64. Anti-Chicken Alexa488 antibody (Invitrogen; A11039)
65. Anti-Chicken Alexa408 antibody (Invitrogen; A48260)
66. Anti-Rabbit Alexa488 antibody (Invitrogen; A21206)
67. Anti-Mouse Alexa555 antibody (Invitrogen; A31570)
68. p-Stat3 Tyr705 antibody (Cell Signaling; 9145T; Clone: D3A7)
69. Tubulin (DSHB; Clone: 4A1)
70. IRDye anti-Rabbit IgG (LI-COR; 926-68071)
71.IRDye anti-Mouse IgG (LI-COR; 926-32210)

Validation

1. Antibody has been validated by manufacturer, RRID:AB_884527. Antibody is for flow analysis of human or mouse cells.
2. Antibody has been validated by manufacturer, RRID:AB_312981. Antibody is for flow analysis of mouse cells.
3. Antibody has been validated by manufacturer, RRID:AB_493731. Antibody is for flow analysis of mouse cells.
4. Antibody has been validated by manufacturer, RRID:AB_893232. Antibody is for flow analysis of mouse cells.
5. Antibody has been validated by manufacturer, RRID:AB_2562415. Antibody is for flow analysis of mouse cells.
6. Antibody has been validated by manufacturer, RRID:AB_11203888. Antibody is for flow analysis of mouse cells.
7. Antibody has been validated by manufacturer, RRID:AB_2214046. Antibody is for flow analysis of mouse cells.
8. Antibody has been validated by manufacturer, RRID:AB_2562622. Antibody is for flow analysis of mouse cells.
9. Antibody has been validated by manufacturer, RRID:AB_2561506. Antibody is for flow analysis of human cells.
10. Antibody has been validated by manufacturer, RRID:AB_314561. Antibody is for flow analysis of human cells.
11-34. Antibodies for CODEX analysis have been validated by manufacturer (https://www.akoyabio.com/phenocycler/assays/). The antibodies are specifically designed for CODEX experiments using human tissue.
36-55. Antibodies for CyTOF analysis have been validated by manufacturer. The antibodies are specifically designed for CyTOF experiments using human cells.
56. Antibody has been validated by manufacturer, RRID:AB_2208679. Antibody is validated for a number of uses and is specific to human or mouse.
57. Antibody has been validated by manufacturer, RRID:AB_10806155. Antibody is validated for a number of uses and is specific to human.
58. Antibody has been validated by manufacturer, RRID:AB_2810223. Antibody is validated for both in vitro and in vivo usage.
59. Antibody has been validated by manufacturer, RRID:AB_35407. Antibody is used for a combination of applications including neutralization.
60. Antibody has been validated by manufacturer, RRID:AB_1107791. The antibody is ideal for use as a non-reative isotype-matched control for mouse IgG2b antibodies in most in vivo applications.
61. Antibody has been validated by manufacturer, RRID:AB_10718041. Antibody is for immunofluorescence of human or mouse tissue.
62. Antibody has been validated by manufacturer, RRID:AB_302459. Antibody is for immunofluorescence of human or mouse tissue.
63. Antibody has been validated by manufacturer. Antibody is for immunofluorescence of human tissue.
64. Antibody has been validated by manufacturer; RRID:AB_2534096. The secondary antibody is used for immunofluorescence.
65. Antibody has been validated by manufacturer; RRID:AB_2890271. The secondary antibody is used for immunofluorescence.
66. Antibody has been validated by manufacturer; RRID:AB_2535792. The secondary antibody is used for immunofluorescence.
67. Antibody has been validated by manufacturer; RRID:AB_2536180. The secondary antibody is used for immunofluorescence.
68. Antibody has been validated by manufacture; RRID:AB_2491009. The antibody is used for western blots using mouse cells.
69. Antibody has been validated by manufacture; RRID:AB_2732839. The antibody is used for western blots using mouse cells.
70. Antibody has been validated by manufacture; RRID:AB_621842. The secondary antibody is used for western blots.
71. Antibody has been validated by manufacture; RRID:AB_10956166. The secondary antibody is used for western blots.

Eukaryotic cell lines

Policy information about cell lines and Sex and Gender in Research

Cell line source(s)

The mouse basal cell carcinoma cell line ASZ001 was generated by Epstein group (So et al., Experimental Dermatology 2006). HEK293-mNoggin-Fc cells were a gift from the Hans Clevers group (Boonekamp et al., PNAS 2019). HEK293-HA-R-Spondin1-Fc (R&D Systems; 3710-001-01) and L-WRN cells (ATCC; CRL-3276) were commercially purchased.

Authentication

ASZ001 were authenticated previously by the Epstein group (So et al., Experimental Dermatology 2006). In our experiments, ASZ001 cells are monitored by their morphology as well, sequencing, and utilization of mouse specific primers. In our experiments, HEK293-mNoggin-Fc, HEK293-HA-R-Spondin1-Fc, and L-WRN cells are monitored by their morphology.

Mycoplasma contamination

No mycoplasma was detected.

Commonly misidentified lines
(See ICLAC register)

None.

Animals and other research organisms

Policy information about studies involving animals; ARRIVE guidelines recommended for reporting animal research, and Sex and Gender in Research

Laboratory animals	Tumors were generated from male or female Ptch1+/-;K14-creER;p53f/f (with or without a RFP reporter). These mice were injected with tamoxifen at 7 weeks of age followed by UV irradiation. Approximately 6-8 months later, tumors would form making the mice 8-10 months of age at tumor collection. 7 week old female C57BL/6J-Tg(UBC-GFP)30Scha/J mice (Jackson Laboratory stock number 004353 and 7-8 week old female NOD SCID (Jackson Laboratory stock number 001303) were used. Mice were housed at an ambient temperature of 72 degrees F with humidity of 42%, with a 12-hour light/dark cycle.
Wild animals	None.
Reporting on sex	Primary tumors were collected from male and female mice without any form of discrimination. No specific consideration was given to use of one particular sex over another. In transplant experiments, female mice were used solely on the basis of mouse cage consolidation (female mice could be housed with one another).
Field-collected samples	None.
Ethics oversight	All mouse usage (husbandry, procedures, experiments, etc.) were approved by the Institutional Animal Care and Use Committee (IACUC) at Stanford University.

Note that full information on the approval of the study protocol must also be provided in the manuscript.

Flow Cytometry

Plots

Confirm that:

- [x] The axis labels state the marker and fluorochrome used (e.g. CD4-FITC).
- [x] The axis scales are clearly visible. Include numbers along axes only for bottom left plot of group (a 'group' is an analysis of identical markers).
- [x] All plots are contour plots with outliers or pseudocolor plots.
- [x] A numerical value for number of cells or percentage (with statistics) is provided.

Methodology

Sample preparation	As noted in methods section, mouse tumors were briefly rinsed with 1x PBS before being chopped and minced into pieces less than 1 mm in diamter. After mincing, tumor pieces are transferred to 50-mL conical tubes and 40 mL of 0.5% collagenase (Gibco; 17-100-017) solution in DKFSM media (Gibco; 10744-019). The minced tumors are then incubated within the solution at 37deg C with rotation for 1 hour. After 1 hour, 5 mL of 0.25% Trypsin (Gibco; 25200056) was then added to the 50-mL conical tube and further incubated for 15 minutes. 5 mL of FBS was then added to the cellular suspension. Cells were filtered with a 70-μm filter before being pelleted. Cells were either immediately used or frozen down in BAM Banker (FujiFilm; CS-02-001). Human tumors were processed in the same manner except that they required a 2 hour collagenase digestion as compared to mice, which only need 1 hour. Samples were incubated at 37°C with rotation for 2 hours. 1 mL of FBS was then added to the cellular suspension. For all flow experiments, samples were re-suspended in a 2% FBS for staining.
Instrument	For flow analysis, we used the LSRII instrument and for sorting, we used the FACSAria II.
Software	BD FACSDiva software was used for data collection on the instrument. For downstream analysis, we used FloJo v10.6.
Cell population abundance	For mouse Cd45+ sorting and analysis experiments, cell viability was approximately 40-70% depending on the tumor, Cd45% ranged from 1-5% depending on the tumor. For human CD45+ analysis experiments, cell viability was approximately 40-70% depending on the tumor, CD45% was approximately 10% depending on the tumor. For mouse Ly6d+ and Ly6d- sorting from primary tumors, the Ly6d+ population made up around 15-30% of the tumor epithelium, depending on the tumor.
Gating strategy	FSC-A/SSC-A gating was first used to identify cells and remove debris. Single cells were isolated by SSC-W/SSC-H and a subsequent FSC-W/FSC-H gating. Live cells were then isolated via negative staining for SytoxBlue. For mouse samples, gating on RFP was done to identifying tumor epithelial cells before Ly6d gating. The boundaries between positive and negative staining were drawn based on a combination of using unstained controls as well as single stain controls.

- [x] Tick this box to confirm that a figure exemplifying the gating strategy is provided in the Supplementary Information.
